# Editorial: Anatomy of Upper Airway and Neuronal Control of Pharyngeal Muscles in Obstructive Sleep Apnea

**DOI:** 10.3389/fneur.2019.00733

**Published:** 2019-07-05

**Authors:** Victor B. Fenik, Thomas Penzel, Atul Malhotra

**Affiliations:** ^1^Department of Medical Research, VA Greater Los Angeles Healthcare System (VHA), Los Angeles, CA, United States; ^2^Websciences International, Los Angeles, CA, United States; ^3^Center of Sleep Medicine, Charité University Hospital, Berlin, Germany; ^4^Division of Pulmonary, Critical Care and Sleep Medicine, University of California, San Diego, San Diego, CA, United States

**Keywords:** upper airway, sleep apnea, loop gain, hypoglossal motoneurons, REM sleep, noradrenergic, cholinergic, control

This Research Topic summarizes the latest findings and new developments in mechanisms of obstructive sleep apnea (OSA) pathophysiology. Many factors contribute to OSA. Both factors, anatomically narrow pharyngeal airway and sleep-related suppression of pharyngeal muscle tone, increase collapsibility of upper airway. In addition, the responsiveness of pharyngeal muscles to negative pressure, low arousal threshold, and high loop gain (unstable ventilatory control in part related to elevated ventilatory chemoreflex control) importantly contribute to OSA (1). The review article by Deacon-Diaz and Malhotra discusses the increase in loop gain by intermittent hypoxia, a hallmark of OSA, and the limitations/challenges of measuring of the loop gain in OSA patients.

In one elegant original paper, a novel non-invasive approach to measuring the collapsibility of upper airway in unanesthetized rodents is described by Nishimura et al. This paper is an important methodological contribution to basic research in OSA pathophysiology, as it describes succinctly methods for studying the control of upper airway muscles during natural sleep and wakefulness.

Upper airway muscles are predominantly innervated and controlled by hypoglossal motoneurons, which receive state-dependent modulatory inputs resulting in the highest motoneuron activity during wakefulness that is decreased during slow wave sleep and maximally suppressed during rapid-eye-movement (REM) sleep. The state-dependent reduction in the activity of hypoglossal motoneurons leads to the sleep-related suppression of upper airway muscle tone, a major non-anatomical contributor to OSA pathophysiology. The original paper by Curado et al. describes the impact of chemogenetic inhibition of hypoglossal motoneurons on the inspiratory flow in mice. The reduction in hypoglossal motoneuron activity markedly increased the occurrence of flow limitation during sleep confirming a critical role of the tone of upper airway muscles in OSA pathophysiology.

The neurotransmitter mechanisms of the suppression of hypoglossal motoneurons during REM sleep have been investigated to help develop pharmacological treatment for OSA. Initially, two major conflicting hypotheses, withdrawal of serotonergic excitation and occurrence of glycinergic inhibition during REM sleep, stimulated studies to uncover these mechanisms. However, direct evidence obtained using different animal models of OSA has suggested that a withdrawal of noradrenergic excitation mediated by α1-adrenoceptors from hypoglossal motoneurons critically contributes to the suppression of their activity during REM sleep (2, 3). In addition, cholinergic inhibition has been suggested to play a major role in the motoneuron depression that was mediated by muscarinic receptors (4). However, the relative effectiveness of these and other neurotransmitters could not be evaluated because the effects of applied antagonists were assessed using different approaches and/or calculations. In the systematic review by Fenik, uniform calculations were applied to all available experimental data obtained using receptor antagonists to assess the involvement of neurotransmitters and their receptors in suppression of the activity of hypoglossal and other motoneurons during REM and non-REM sleep. One major finding of these calculations suggests that the relative contribution of the withdrawal of noradrenergic excitation and cholinergic (muscarinic) inhibition to the depression of hypoglossal motoneuron activity during REM sleep is ~50% each; whereas glycinergic or serotonergic drives have negligible or no contribution to the effect of sleep on hypoglossal motoneurons.

A recent proof-of-concept trial on unselected OSA patients demonstrates that it is possible to improve OSA significantly at least for one night by systemic administration of noradrenaline reuptake inhibitor atomoxetine in combination with a muscarinic antagonist oxybutynin (5). This finding is a notable example of a high translational value of the knowledge that is accumulated in basic research and applied to the clinical trials. It also gives motivation to go forward with more detailed studies that will focus on modulatory inputs to both noradrenergic and cholinergic neural pathways to hypoglossal motoneurons, which will provide information for additional options of pharmacological treatment of OSA. A thorough review by Rukhadze and Fenik of the neuroanatomical data of the noradrenergic and cholinergic control of state-dependent activity of hypoglossal motoneurons provides groundwork for such studies. Only through further work into basic mechanisms underlying OSA disease pathogenesis new therapeutic targets are likely to emerge.

## Author Contributions

All authors listed have made a substantial, direct and intellectual contribution to the work, and approved it for publication.

### Conflict of Interest Statement

The authors declare that the research was conducted in the absence of any commercial or financial relationships that could be construed as a potential conflict of interest.

## References

[1] Eckert DJ White DP Jordan AS Malhotra A Wellman A. Defining phenotypic causes of obstructive sleep apnea. Identification of novel therapeutic targets. Am J Respir Crit Care Med. (2013) 188:996–1004. 10.1164/rccm.201303-0448OC 23721582 PMC3826282

[2] Fenik V Davies R Kubin L. REM sleep-like atonia of hypoglossal (XII) motoneurons is caused by loss of noradrenergic and serotonergic inputs. Am J Respir Crit Care Med. (2005) 172:1322–30. 10.1164/rccm.200412-1750OC 16100007 PMC5222563

[3] Chan E Steenland H Liu H Horner R. Endogenous excitatory drive modulating respiratory muscle activity across sleep-wake states. Am J Respir Crit Care Med. (2006) 174:1264–73. 10.1164/rccm.200605-597OC 16931636

[4] Grace KP Hughes SW Horner RL Identification of the mechanism mediating genioglossus reactivation muscle suppression in REM sleep. Am J Respir Crit Care Med. (2013) 187:311–9. 10.1164/rccm.201209-1654OC 23220910

[5] Taranto-Montemurro L Messineo L Sands SA Azarbarzin A Marques M Edwards BA. The combination of atomoxetine and oxybutynin greatly reduces obstructive sleep apnea severity: a randomized, placebo-controlled, double-blind crossover trial. Am J Respir Crit Care Med. (2018) 199:1267–76. 10.1164/rccm.201808-1493OC 30395486 PMC6519859

